# Real-World Outcomes of Oral VEGFR Tyrosine Kinase Inhibitor-Containing Therapy in Unresectable Malignant Mesothelioma: A Single-Center Retrospective Study

**DOI:** 10.3390/cancers18213541

**Published:** 2026-11-04

**Authors:** Zheng Liu, Jia Zhong, Rui Wan, Jian-Chun Duan, Zhi-Jie Wang, Hua Bai, Jie Wang

**Affiliations:** State Key Laboratory of Molecular Oncology, Beijing Key Laboratory, CAMS Key Laboratory of Translational Research on Lung Cancer, Department of Medical Oncology, National Cancer Center/National Clinical Research Center for Cancer/Cancer Hospital, Chinese Academy of Medical Sciences and Peking Union Medical College, Beijing 100021, China

**Keywords:** malignant mesothelioma, VEGFR tyrosine kinase inhibitor, anlotinib, apatinib, retrospective study, progression-free survival

## Abstract

Background: Systemic treatment choices after failure of standard therapy are limited for malignant mesothelioma. Oral vascular endothelial growth factor receptor tyrosine kinase inhibitors (VEGFR-TKIs) have been incorporated into salvage treatment strategies, but real-world evidence regarding their effectiveness and safety remains scarce. Methods: We retrospectively analyzed a single-center cohort comprising patients with pathologically confirmed malignant mesothelioma treated at Cancer Hospital, Chinese Academy of Medical Sciences/National Cancer Center between 1 April 2017 and 16 March 2023. Patients with unresectable disease who received a VEGFR-TKI-containing regimen and had sufficient clinical follow-up for outcome assessment were included. Progression-free survival (PFS) served as the primary endpoint. Objective response rate (ORR), disease control rate (DCR), overall survival (OS) from VEGFR-TKI initiation, and recorded adverse events were included as secondary endpoints. Results: The cohort comprised 48 patients, with a median follow-up of 24.0 months. The Kaplan–Meier estimated median PFS was 4.8 months (95% CI, 2.9–8.8), and median OS from VEGFR-TKI initiation was 17.9 months (95% CI, 13.5–not estimable). Among 39 response-evaluable patients, 6 achieved partial response, 20 had stable disease, and 13 had progressive disease as their best overall response, yielding an ORR of 15.4% and a DCR of 66.7%. Two patients remained progression-free for more than 24 months. Grade 3 adverse events in the records included hematuria, hypertension, and nausea/vomiting; no grade 4 or grade 5 adverse event was documented. Conclusions: VEGFR-TKI-containing therapy was associated with clinically relevant disease control in unresectable malignant mesothelioma, with durable benefit observed in a small subset of patients. These observations warrant further evaluation of VEGFR-TKI-based salvage strategies in larger multicenter cohorts.

## Simple Summary

Malignant mesothelioma is an uncommon and aggressive cancer for which few treatment options remain once standard therapy has failed. Oral medicines that interfere with signals involved in tumor blood vessel formation have been used in clinical practice, but evidence supporting their use in mesothelioma remains limited. We retrospectively evaluated 48 patients with unresectable malignant mesothelioma who received treatment containing these drugs at a single cancer center. The median time before progression was 4.8 months, and the median overall survival after treatment initiation was 17.9 months. Among patients whose tumors could be evaluated, approximately two-thirds achieved a partial response or stable disease, and a small subset experienced prolonged disease control. Severe treatment-related adverse events were uncommon in the available records. These findings provide real-world evidence supporting further investigation of these treatments in larger studies.

## 1. Introduction

Malignant mesothelioma is an uncommon malignancy of mesothelial-lined serosal surfaces, with the pleura and peritoneum representing the predominant sites. According to GLOBOCAN 2022, mesothelioma comprised approximately 0.15% of incident cancers worldwide [1]. The prognosis of malignant mesothelioma remains poor, with a reported median overall survival of approximately 12 months [2]. Limited salvage options are available for malignant mesothelioma after failure of first-line systemic therapy, which is generally based on platinum plus pemetrexed-containing regimens.

Mesothelioma cells can secrete VEGF ligands and express VEGF-related receptors, supporting paracrine and autocrine VEGF signaling within the tumor microenvironment [3,4,5]. These observations support angiogenesis as a therapeutic target in mesothelioma. Randomized evidence and guideline recommendations support bevacizumab plus pemetrexed/platinum as a first-line option for eligible patients with unresectable malignant pleural mesothelioma [6,7]. However, evidence for oral VEGFR-TKIs in mesothelioma remains less established. Phase II studies of cediranib, sunitinib, vatalanib, and pazopanib generally reported modest objective response rates with disease stabilization in certain patients [8,9,10,11,12]. The improvement in PFS observed with nintedanib plus cisplatin/pemetrexed in the phase II LUME-Meso trial was not reproduced in the subsequent phase III study [13,14].

Anlotinib and apatinib have been used as oral VEGFR-TKIs in patients with refractory mesothelioma in real-world practice. Anlotinib inhibits multiple kinases, including VEGFR, FGFR and PDGFR, whereas apatinib primarily targets VEGFR2 [15,16]. In a 27-patient pilot study of apatinib for refractory diffuse malignant peritoneal mesothelioma, the reported ORR and DCR were 14.8% and 59.3%, respectively; median apatinib-treatment-related survival was 14.0 months and median OS was 59.4 months [17]. Additional published evidence for apatinib- or anlotinib-containing regimens in mesothelioma remains mainly case-based, with several reports describing prolonged disease control and PFS ranging from 10 to 24 months [18,19,20,21]. These observations suggest potential activity of VEGFR-TKIs in mesothelioma, but their efficacy and safety remain insufficiently characterized because cohort-level real-world data are scarce.

To address this evidence gap, we retrospectively evaluated consecutive patients with unresectable malignant mesothelioma who received oral VEGFR-TKI-containing regimens after prior systemic therapy at our center, focusing on efficacy and safety outcomes.

## 2. Materials and Methods

### 2.1. Study Design and Patients

Institutional records were retrospectively reviewed to identify consecutive patients with pathologically confirmed malignant mesothelioma treated at Cancer Hospital, Chinese Academy of Medical Sciences/National Cancer Center from 1 April 2017 to 16 March 2023. Eligibility required all of the following: (1) pathologically confirmed malignant mesothelioma; (2) unresectable disease or disease not amenable to surgery; (3) receipt of a VEGFR-TKI-containing regimen; and (4) follow-up data available after VEGFR-TKI initiation. Exclusion criteria included: (1) insufficient treatment documentation for treatment classification; (2) no documented VEGFR-TKI exposure; (3) incomplete VEGFR-TKI treatment records; or (4) insufficient follow-up for survival or response assessment. Variables were coded as unknown when the relevant information was not documented in the available medical records. Histologic subtype was coded as unknown when the archival pathology records did not contain sufficient information for reliable subtyping.

The Ethics Committee of the National Cancer Center/Cancer Hospital, Chinese Academy of Medical Sciences (approval No. 26/215-5939; approval date: 1 April 2026) granted ethics approval for this study and waived the requirement for informed consent owing to the retrospective design of the study.

### 2.2. Treatment Exposure, Response Assessment, and Endpoints

VEGFR-TKI-containing regimens were administered as monotherapy or combined with chemotherapy, immune checkpoint blockade (ICB), or both. In the final analytic cohort, all evaluable VEGFR-TKI exposures involved either anlotinib or apatinib. Systemic therapies were administered according to standard recommended dosing schedules in routine clinical practice. Radiologic response was assessed according to disease site: pleural disease was evaluated according to modified RECIST 1.1 for mesothelioma using perpendicular measurements of pleural tumor thickness, whereas peritoneal disease was evaluated according to standard RECIST 1.1 using unidimensional measurements of target lesions [22,23]. Available CT or MRI examinations were reviewed independently by two investigators (Z.L. and R.W.), and any discrepancies were resolved by consensus after joint review. Best overall response was classified into complete response, partial response, stable disease, or progressive disease. Patients without sufficient imaging data for formal radiologic response assessment were not assigned a best overall response and were excluded from the ORR and DCR calculations. These patients remained included in the PFS and OS analyses when progression, death, or censoring could be determined from the available clinical follow-up.

For each patient, only the first VEGFR-TKI treatment course was included in the main efficacy analysis to avoid potential bias from cross-line VEGFR-TKI treatment.

PFS, the primary endpoint, was measured from VEGFR-TKI initiation until radiologic or clinical progression or death from any cause. Secondary endpoints were ORR, DCR, OS from VEGFR-TKI initiation, and recorded adverse events. Adverse events captured from clinical notes, treatment records, and laboratory data were assigned grades based on the Common Terminology Criteria for Adverse Events (CTCAE) version 5.0 [24].

### 2.3. Statistical Analysis

Categorical variables were described using counts and percentages, whereas continuous variables were summarized by the median and interquartile range (IQR), as appropriate. Survival was estimated with the Kaplan–Meier method. Exploratory between-group comparisons were performed using log-rank tests. Univariable Cox proportional hazards regression models examined associations of selected clinical variables with PFS or OS. Multivariable Cox modeling was omitted because of the modest cohort size and limited number of events, which increased the risk of model overfitting and unstable estimates. Pearson correlation analysis was used to examine the association between PFS on prior bevacizumab and PFS on subsequent VEGFR-TKI therapy. All *p* values were two-sided. *p* < 0.05 was regarded as nominally significant, without adjustment for multiple comparisons. Analyses were conducted in R (version 4.2.2) and GraphPad Prism 8. Efficacy outcomes were also summarized descriptively according to VEGFR-TKI treatment pattern (monotherapy, combination with ICB, combination with chemotherapy, or combination with both chemotherapy and ICB). Given the small and uneven subgroup sizes and non-random treatment allocation, these analyses were not intended for formal comparative inference.

## 3. Results

### 3.1. Cohort Selection and Baseline Characteristics

A total of 833 pathologically confirmed mesothelioma cases were screened. Treatment documentation was insufficient in 563 cases, and 206 had no documented VEGFR-TKI exposure. Among the remaining 64 patients with documented VEGFR-TKI exposure who underwent detailed chart review, 16 were excluded because evaluable follow-up after VEGFR-TKI initiation was unavailable, leaving 48 patients in the final analytic population (Figure 1). PFS and OS were analyzed in all 48 patients. Radiologic response analyses, including ORR and DCR, were restricted to 39 patients with sufficient imaging data for formal radiologic response assessment. In the remaining nine patients, the imaging data required for formal retrospective radiologic response assessment were unavailable for review.

Table 1 summarizes baseline clinical and disease characteristics. Median age at diagnosis was 59.0 years (IQR, 51.6–63.6 years), and median age at VEGFR-TKI initiation was 59.7 years (IQR, 52.0–65.2 years). The cohort comprised 25 males (52.1%) and 23 females (47.9%). Four patients (8.3%) reported a definite history of asbestos exposure, and 17 (35.4%) had a history of long-term heavy smoking. Pleural disease was present in 36 patients (75.0%), whereas 12 patients (25.0%) had peritoneal disease.

Histologic subtype was documented in 30 patients. In the remaining 18 patients, the archival pathology records did not contain sufficient information to determine the histologic subtype reliably. Epithelioid mesothelioma was observed in 22 patients, biphasic mesothelioma in 5, and sarcomatoid mesothelioma in 3. Three patients (6.3%) had BAP1-associated malignancies, including breast cancer and renal cancer. Twelve patients (25.0%) had a family history of cancer.

Prior treatment exposures are summarized in Table 2. All patients had previously received systemic therapy before VEGFR-TKI initiation. The median number of prior systemic therapy lines was 2 (range, 1–5). Twenty-nine patients (60.4%) had previously received anti-angiogenic therapy, including bevacizumab in 26 patients (54.2%).

### 3.2. Treatment Patterns of VEGFR-TKI-Containing Regimens

Detailed regimen combinations are provided in Table S1 in the Supplementary Materials. VEGFR-TKI-containing therapy was administered as monotherapy in 18 patients (37.5%), with ICB in 6 (12.5%), with chemotherapy in 13 (27.1%), and with both chemotherapy and ICB in 11 (22.9%). Anlotinib was used in 36 patients (75.0%) and apatinib in 12 patients (25.0%).

### 3.3. Efficacy Outcomes

In the analytic cohort of 48 patients, the median follow-up was 24.0 months. Median PFS was 4.8 months (95% CI, 2.9 to 8.8), and median OS from VEGFR-TKI initiation was 17.9 months (95% CI, 13.5 to NE). Figure 2A,B display the Kaplan–Meier estimates of PFS and OS from treatment initiation, respectively.

Among the 39 response-evaluable patients, 6 (15.4%) achieved PR, 20 (51.3%) had SD, and 13 (33.3%) had PD; no CR was observed. The ORR was 15.4%, and the DCR was 66.7%. The distribution of BOR and best percentage change in target lesions is shown in the waterfall plot (Figure 3A).

Descriptive efficacy outcomes stratified by VEGFR-TKI treatment pattern are summarized in Table 3. Because of the small and uneven subgroup sizes and non-random treatment allocation, these results were not used to infer comparative efficacy or the independent contribution of individual treatment components.

Six patients achieved PR after their first VEGFR-TKI treatment course, and their clinicopathologic characteristics and treatment courses are shown in the swimmer plot (Figure 3B). Among them, four were male, four had pleural mesothelioma, four received an anlotinib-containing regimen, and four had previously received anti-angiogenic therapy. Two of the six patients with partial response had PFS durations exceeding 24 months. One patient with pleural mesothelioma received apatinib plus pemetrexed and achieved a PFS of 30.0 months. The other patient with epithelioid peritoneal mesothelioma received apatinib monotherapy and remained progression-free for 55.0 months.

### 3.4. Exploratory Analyses

Exploratory subgroup survival analyses and univariable Cox regression were performed to assess associations between baseline or treatment-related factors and PFS or OS. In exploratory analyses, patients receiving apatinib-containing regimens had a median PFS of 7.9 months compared with 4.0 months for patients receiving anlotinib-containing regimens (log-rank *p* = 0.038). The corresponding univariable HR was 0.46 (95% CI, 0.21–0.98; *p* = 0.043). Baseline and treatment characteristics according to VEGFR-TKI agent are summarized in Supplementary Table S4. Given the small apatinib subgroup and between-group differences in clinical and treatment characteristics, this unadjusted finding should be considered exploratory and hypothesis-generating. None of the other evaluated baseline or treatment-related variables was significantly associated with PFS or OS. Detailed subgroup survival and univariable Cox regression results are provided in Tables S2 and S3 in the Supplementary Materials.

In an exploratory Pearson correlation analysis, no correlation between prior bevacizumab PFS and subsequent VEGFR-TKI PFS was observed in this cohort (Pearson r = 0.084, *p* = 0.684).

### 3.5. Safety Profile

Adverse events during VEGFR-TKI-containing therapy are listed in Table 4. Hypertension was recorded most frequently (14.6%), followed by increased creatinine and leukopenia (8.3% each). Proteinuria and rash were recorded in 3 patients each (6.3%), whereas anemia and oral ulcers were recorded in 2 patients each (4.2%). Hematuria, hemoptysis, and nausea/vomiting were each recorded in 1 patient (2.1%). Documented grade 3 adverse events included hematuria, hypertension, and nausea/vomiting; no grade 4 or grade 5 adverse events were identified. Two patients discontinued VEGFR-TKI-containing therapy because of grade 3 adverse events, including hypertension in one patient and nausea/vomiting in another patient.

## 4. Discussion

In our single-center retrospective cohort of patients with unresectable malignant mesothelioma, VEGFR-TKI-containing therapy was associated with clinical activity. Median PFS and OS from VEGFR-TKI initiation were 4.8 and 17.9 months, respectively. Among response-evaluable patients, the ORR was 15.4% and the DCR was 66.7%. Previous phase II studies of oral antiangiogenic TKIs in mesothelioma have generally reported objective response rates of approximately 6–12% and stable disease in approximately 34–72% of patients [8,10,11,12]. Among studies reporting PFS, median PFS was approximately 2.6–4.2 months [8,11,12]. In a pilot study of apatinib for refractory diffuse malignant peritoneal mesothelioma, the ORR and DCR were 14.8% and 59.3%, respectively [17]. In comparison, our cohort showed an ORR of 15.4%, stable disease in 51.3% of response-evaluable patients, a DCR of 66.7%, and a median PFS of 4.8 months. Taken together, our findings are consistent with several earlier studies, in which objective responses were generally limited to a minority of patients, whereas stable disease was more frequently observed. These cross-study comparisons should be interpreted cautiously because of differences in disease site, treatment line, study design, and treatment regimens. These findings indicate that the clinical value of VEGFR-TKI-containing therapy may lie in disease control rather than frequent tumor shrinkage in refractory mesothelioma. Our study therefore adds real-world evidence for oral VEGFR-TKIs in a rare tumor setting where effective systemic options remain limited.

A small subset of patients experienced prolonged benefit. Six patients achieved PR after their first VEGFR-TKI treatment course, and two of them maintained disease control for more than two years. These cases highlight the heterogeneity of clinical benefit from VEGFR-TKI-containing therapy and support further research to identify clinical or biological markers of durable response.

Exploratory analyses also provided several hypothesis-generating observations. The exploratory association between apatinib-containing therapy and longer PFS should be interpreted cautiously. The comparison was unadjusted, involved only 12 apatinib-treated patients, and the two treatment groups differed in several baseline and treatment characteristics, including primary site, prior treatment exposure, and concomitant therapy (Table S4). Therefore, this finding warrants further evaluation in larger cohorts and should not be considered evidence of comparative superiority of apatinib over anlotinib. In addition, Pearson correlation analysis indicated no significant correlation between prior bevacizumab PFS and subsequent VEGFR-TKI PFS in this cohort, a finding suggesting that prior benefit from bevacizumab-based therapy should not be used as the sole criterion to determine whether VEGFR-TKIs should be applied later.

The observed adverse-event profile comprised vascular, renal or urinary, hematologic, gastrointestinal, and mucocutaneous toxicities. Grade 3 adverse events were infrequent, and no grade 4 or grade 5 adverse events were identified. However, because adverse-event ascertainment was based on retrospective review of routine clinical documentation rather than protocol-specified prospective toxicity monitoring, the frequency of grade 1–2 adverse events and toxicities managed in the outpatient setting may have been underestimated.

These findings should be interpreted in light of several constraints. First, the retrospective design creates the potential for selection bias, and reliance on existing clinical documentation may also introduce information bias in efficacy and safety ascertainment. Histologic subtype could not be determined in 18 patients because of incomplete archival pathology information, limiting assessment of histology-related prognostic heterogeneity and potentially contributing to residual confounding in the survival analyses. Second, the modest cohort size, reflecting both the rarity of mesothelioma and the uncommon use of VEGFR-TKI-containing therapy in this setting, limited statistical power for the log-rank, Cox, and Pearson correlation analyses. These analyses are therefore exploratory and should be interpreted with caution. Nine patients lacked sufficient imaging data for formal radiologic response assessment and were therefore excluded from the ORR and DCR analyses. Review of the available PFS data showed that no PFS event was documented before the usual time point for the first radiologic response assessment in eight of the nine patients; the remaining patient was censored earlier without a documented progression event. Their best overall responses could nevertheless not be determined reliably. Restricting radiologic response assessment to the 39 response-evaluable patients may therefore have introduced attrition-related selection bias, and the ORR and DCR estimates should be interpreted with appropriate caution. Finally, substantial treatment heterogeneity limited the interpretation of efficacy outcomes. Because VEGFR-TKIs were administered either as monotherapy or in combination with chemotherapy, ICB, or both, the observed outcomes cannot be attributed solely to the VEGFR-TKI component. The treatment-pattern subgroup analyses should therefore be considered descriptive and should not be interpreted as comparative evidence among treatment strategies. Differences in prior treatment history, including prior surgical treatment, may also have contributed to prognostic heterogeneity. As overall survival may have been influenced by heterogeneous post-progression anticancer therapies, the observed OS should be considered descriptive and should not be attributed solely to VEGFR-TKI-containing therapy. Further prospective studies are required to validate these findings.

## 5. Conclusions

In this single-center retrospective cohort, VEGFR-TKI-containing regimens were associated with disease control in patients with unresectable malignant mesothelioma. A small subset of patients achieved durable benefit, and adverse events appeared manageable. Larger multicenter studies are warranted to further explore the efficacy and safety of VEGFR-TKI-containing therapy as a salvage option and to determine the patients who are most likely to derive benefit.

## Figures and Tables

**Figure 1:**
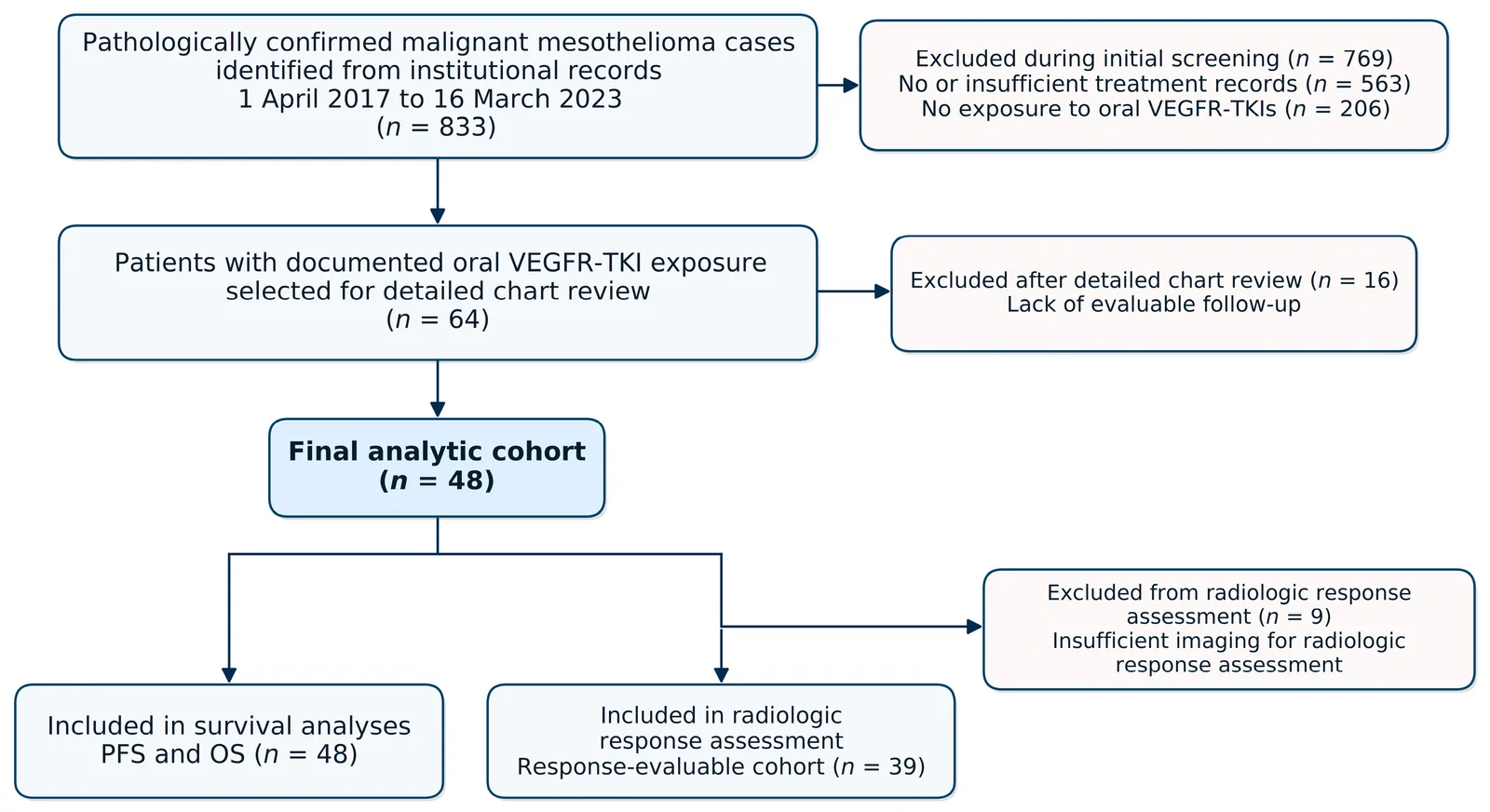
Cohort assembly and analysis populations. Pathologically confirmed malignant mesothelioma cases were identified from institutional records between 1 April 2017 and 16 March 2023. The final analytic cohort included 48 patients with documented oral VEGFR-TKI-containing therapy and evaluable follow-up. All 48 patients were included in the survival analyses, and 39 patients with post-baseline imaging available for independent radiologic response assessment were included in the response-evaluable population. OS, overall survival; PFS, progression-free survival; VEGFR-TKI, vascular endothelial growth factor receptor tyrosine kinase inhibitor.

**Figure 2:**
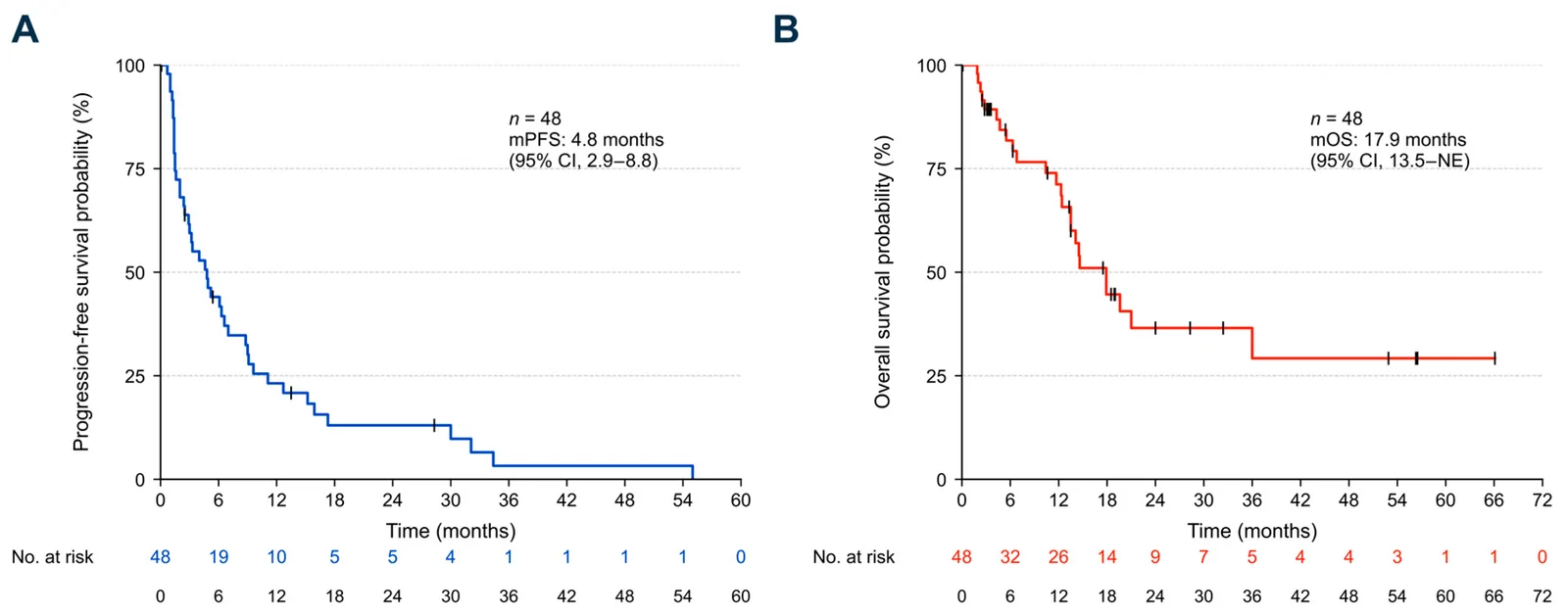
Kaplan–Meier estimates of progression-free survival and overall survival. (**A**) PFS from initiation of oral VEGFR-TKI therapy in the final analytic cohort; (**B**) OS from initiation of oral VEGFR-TKI therapy in the final analytic cohort. Censored observations are marked by ticks, with risk-set counts displayed beneath each plot. CI, confidence interval; mOS, median overall survival; mPFS, median progression-free survival; NE, not estimable; OS, overall survival; PFS, progression-free survival; VEGFR-TKI, vascular endothelial growth factor receptor tyrosine kinase inhibitor.

**Figure 3:**
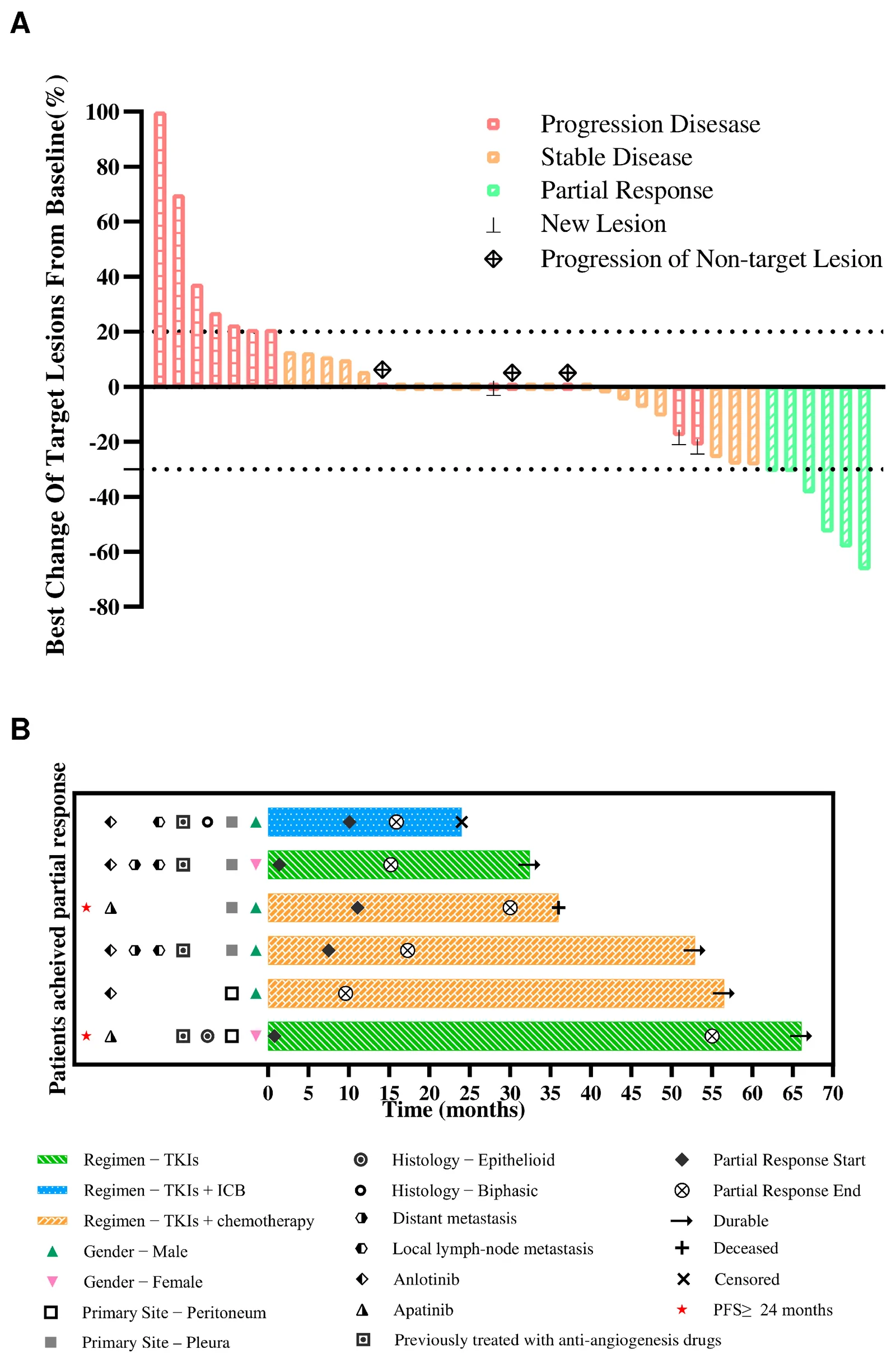
Radiologic response and clinical course of patients achieving partial response. (**A**) Waterfall representation of the maximum percentage change in target-lesion size from baseline among the 39 response-evaluable patients. Response categories reflect overall radiologic assessment, including target lesions, non-target lesions, and new lesions. The dotted horizontal lines indicate the −30% threshold for partial response (PR) and the +20% reference level for progressive disease (PD); (**B**) swimmer plot showing treatment duration and response course among the six patients achieving partial response. ICB, immune checkpoint blockade; PFS, progression-free survival; PR, partial response; RECIST, Response Evaluation Criteria in Solid Tumors; TKI, tyrosine kinase inhibitor.

**Table 1:** Patient and disease characteristics.

Characteristic	Overall Cohort (*N* = 48)
Age at diagnosis, years	59.0 [51.6–63.6]
Age at VEGFR-TKI initiation, years	59.7 [52.0–65.2]
Sex	
Male	25 (52.1)
Female	23 (47.9)
Primary site	
Pleura	36 (75.0)
Peritoneum	12 (25.0)
Histologic subtype	
Epithelioid	22 (45.8)
Biphasic	5 (10.4)
Sarcomatoid	3 (6.3)
Unknown	18 (37.5)
Regional lymph node metastasis	
Yes	24 (50.0)
No	23 (47.9)
Uncertain	1 (2.1)
Distant organ metastasis	
Yes	25 (52.1)
No	23 (47.9)
Asbestos exposure	
Yes	4 (8.3)
No	36 (75.0)
Unknown	8 (16.7)
Smoking history	
Yes	17 (35.4)
No	24 (50.0)
Unknown	7 (14.6)
Family history of cancer	
Yes	12 (25.0)
No	29 (60.4)
Unknown	7 (14.6)
BAP1-associated malignancy history	
Yes	3 (6.3)
No	43 (89.6)
Unknown	2 (4.2)
VEGFR-TKI agent	
Anlotinib	36 (75.0)
Apatinib	12 (25.0)

Data are presented as *n* (%) or median [IQR]. Percentages are based on the overall analytic cohort (*N* = 48). IQR, interquartile range; VEGFR-TKI, vascular endothelial growth factor receptor tyrosine kinase inhibitor. Unknown histologic subtype indicates that the archival pathology records did not contain sufficient information to determine the histologic subtype reliably. Unknown asbestos exposure or smoking history indicates that the corresponding information was not documented in the available medical records.

**Table 2:** Prior treatment exposure and VEGFR-TKI treatment patterns.

Treatment Characteristic	Overall Cohort (*N* = 48)
Prior cytoreductive surgery	
Yes	5 (10.4)
No	43 (89.6)
Prior systemic therapy lines	
Median (range)	2 (1–5)
≤2 lines	38 (79.2)
>2 lines	10 (20.8)
Prior anti-angiogenic therapy	
Yes	29 (60.4)
No	19 (39.6)
Prior bevacizumab exposure	
Yes	26 (54.2)
No	22 (45.8)
Cross-line VEGFR-TKI exposure	
Yes	8 (16.7)
No	40 (83.3)
VEGFR-TKI agent	
Anlotinib	36 (75.0)
Apatinib	12 (25.0)
VEGFR-TKI treatment pattern	
Monotherapy	18 (37.5)
With ICB	6 (12.5)
With chemotherapy	13 (27.1)
With chemotherapy plus ICB	11 (22.9)

Data are presented as *n* (%). Percentages are based on the overall analytic cohort (*N* = 48). ICB, immune checkpoint blockade; VEGFR-TKI, vascular endothelial growth factor receptor tyrosine kinase inhibitor.

**Table 3:** Efficacy outcomes according to VEGFR-TKI treatment pattern.

Treatment Pattern	*N*	Response-Evaluable *n*	ORR, *n/N* (%)	DCR, *n/N* (%)	Median PFS, Months (95% CI)	Median OS, Months (95% CI)
VEGFR-TKI monotherapy	18	14	2/14 (14.3)	10/14 (71.4)	4.6 (2.9–12.7)	17.9 (12.3–NE)
VEGFR-TKI + ICB	6	5	1/5 (20.0)	2/5 (40.0)	2.0 (1.4–NE)	14.1 (13.5–NE)
VEGFR-TKI + chemotherapy	13	13	3/13 (23.1)	11/13 (84.6)	6.6 (1.6–NE)	14.6 (6.3–NE)
VEGFR-TKI + chemotherapy + ICB	11	7	0/7 (0.0)	3/7 (42.9)	2.5 (1.4–NE)	14.5 (4.3–NE)

ORR and DCR were calculated among response-evaluable patients within each treatment-pattern subgroup. Median PFS and OS and their 95% confidence intervals were estimated using the Kaplan–Meier method. These subgroup analyses are descriptive because of the small and uneven subgroup sizes and non-random treatment allocation. CI, confidence interval; DCR, disease control rate; ICB, immune checkpoint blockade; NE, not estimable; ORR, objective response rate; OS, overall survival; PFS, progression-free survival; VEGFR-TKI, vascular endothelial growth factor receptor tyrosine kinase inhibitor.

**Table 4:** Adverse events during VEGFR-TKI-containing therapy.

Adverse Event	Grade 1	Grade 2	Grade 3	Grade 4	Grade 5	Any Grade, *n/N* (%)
Hypertension	2	4	1	0	0	7/48 (14.6)
Creatinine increased	0	4	0	0	0	4/48 (8.3)
Leukopenia	3	1	0	0	0	4/48 (8.3)
Proteinuria	0	3	0	NA	NA	3/48 (6.3)
Rash	3	0	0	NA	NA	3/48 (6.3)
Anemia	2	0	0	0	0	2/48 (4.2)
Oral ulcers	1	1	0	0	0	2/48 (4.2)
Hematuria	0	0	1	0	0	1/48 (2.1)
Hemoptysis	1	0	0	0	0	1/48 (2.1)
Nausea/vomiting	0	0	1	0	0	1/48 (2.1)

Adverse events were identified retrospectively from available clinical documentation and laboratory records. Values indicate the number of patients with adverse events. Percentages are based on the overall analytic cohort (*N* = 48). Categories are not mutually exclusive. Adverse events were coded and graded according to CTCAE version 5.0 when sufficient information was available. NA indicates that the corresponding grade is not defined for the specific CTCAE term. CTCAE, Common Terminology Criteria for Adverse Events; VEGFR-TKI, vascular endothelial growth factor receptor tyrosine kinase inhibitor.

## Data Availability

The data presented in this study are available from the corresponding author on reasonable request and subject to institutional approval. They are not publicly available because they contain institutional clinical data and potentially identifiable patient information.

## Supplementary Materials

The following supporting information can be downloaded at: https://www.mdpi.com/article/10.3390/cancers18213541/s1, Additional file S1: Supplementary Tables S1–S4. File format: DOCX. Description: Supplementary Table S1. Treatment regimen components; Supplementary Table S2. Subgroup Kaplan-Meier estimates and log-rank tests; Supplementary Table S3. Univariable Cox regression analyses for PFS and OS; Supplementary Table S4. Baseline and treatment characteristics according to VEGFR-TKI agent.

## Abbreviations

The following abbreviations are used in this manuscript:

BOR	best overall response
CI	confidence interval
CT	computed tomography
CTCAE	Common Terminology Criteria for Adverse Events
DCR	disease control rate
ICB	immune checkpoint blockade
IQR	interquartile range
MRI	magnetic resonance imaging
ORR	objective response rate
OS	overall survival
PD	progressive disease
PFS	progression-free survival
PR	partial response
RECIST	Response Evaluation Criteria in Solid Tumors
SD	stable disease
VEGFR-TKI	vascular endothelial growth factor receptor tyrosine kinase inhibitor
