# Supplementary material for: Real-World Outcomes of Oral VEGFR Tyrosine Kinase Inhibitor-Containing Therapy in Unresectable Malignant Mesothelioma: A Single-Center Retrospective Study
Source: Cancers (Basel). 2026 Nov 4;18(21):3541. doi: 10.3390/cancers18213541 (PMC13762079; doi:10.3390/cancers18213541)
Supplement: Supplementary file 1 [file cancers-18-03541-s001.zip › cancers-4536910-supplementary.pdf]

Additional file S1: Supplementary Tables

Supplementary tables for: Real-world outcomes of oral VEGFR tyrosine kinase inhibitor-containing therapy in unresectable malignant mesothelioma: a single-center retrospective study

Supplementary Table S1. Treatment regimen components

Regimen component	*n* (%)
**VEGFR-TKI treatment pattern**	
VEGFR-TKI monotherapy	18 (37.5)
VEGFR-TKI + ICB	6 (12.5)
VEGFR-TKI + chemotherapy	13 (27.1)
VEGFR-TKI + chemotherapy + ICB	11 (22.9)
**VEGFR-TKI agent**	
Anlotinib	36 (75.0)
Apatinib	12 (25.0)
**Chemotherapy agent in combination regimens**	
Pemetrexed	8 (33.3)
Nab-paclitaxel	7 (29.2)
Gemcitabine	7 (29.2)
Irinotecan	1 (4.2)
Paclitaxel	1 (4.2)
**ICB agent in combination regimens**	
Sintilimab	8 (47.1)
Pembrolizumab	4 (23.5)
Tislelizumab	2 (11.8)
Nivolumab	1 (5.9)
Nivolumab + ipilimumab	1 (5.9)
PD-1 inhibitor, not specified	1 (5.9)

Percentages for VEGFR-TKI treatment pattern and VEGFR-TKI agent are based on the full cohort ($N = 48$). Percentages for chemotherapy and ICB agents are based on patients receiving the corresponding combination component. ICB, immune checkpoint blockade; VEGFR-TKI, vascular endothelial growth factor receptor tyrosine kinase inhibitor. "PD-1 inhibitor, not specified" indicates that treatment with a PD-1 inhibitor was documented, but the specific agent was not recorded in the available medical records.

Supplementary Table S2. Subgroup Kaplan-Meier estimates and log-rank tests

Variable	Group (*n*)	Median PFS, months (95% CI)	PFS log-rank *p* value	Median OS, months (95% CI)	OS log-rank *p* value
Age at VEGFR-TKI initiation	≥60 yr (*n* = 24)<br><60 yr (*n* = 24)	6.1 (3.0–9.1)<br>3.2 (1.6–17.3)	0.631	14.6 (12.3–NE)<br>17.9 (12.4–NE)	0.377
Sex	Male (*n* = 25)<br>Female (*n* = 23)	3.3 (2.5–9.6)<br>5.2 (2.4–15.2)	0.561	14.6 (10.4–NE)<br>17.9 (13.5–NE)	0.408
Primary site	Peritoneal (*n* = 12)<br>Pleural (*n* = 36)	7.9 (2.4–NE)<br>4.0 (2.5–7.0)	0.104	14.6 (6.3–NE)<br>17.9 (13.5–NE)	0.936
Histology	Non-epithelioid (*n* = 8)<br>Epithelioid (*n* = 22)	6.6 (1.4–NE)<br>3.2 (2.4–11.1)	0.843	19.6 (17.9–NE)<br>14.6 (12.4–NE)	0.348
Regional lymph node metastasis	Yes (*n* = 24)<br>No (*n* = 23)	4.8 (3.2–15.2)<br>4.6 (2.0–9.6)	0.699	14.6 (6.8–NE)<br>17.9 (12.4–NE)	0.461

Variable	Group (*n*)	Median PFS, months (95% CI)	PFS log-rank *p* value	Median OS, months (95% CI)	OS log-rank *p* value
Distant organ metastasis	Yes (*n* = 25)<br>No (*n* = 23)	4.0 (2.0–9.1)<br>4.9 (3.2–12.7)	0.287	14.5 (11.7–NE)<br>19.6 (14.1–NE)	0.380
Lung metastasis	Yes (*n* = 11)<br>No (*n* = 37)	5.2 (1.5–NE)<br>4.7 (2.5–9.1)	0.777	17.9 (14.5–NE)<br>14.6 (12.4–NE)	0.506
Liver metastasis	Yes (*n* = 3)<br>No (*n* = 45)	2.4 (1.4–NE)<br>4.8 (3.0–9.1)	0.222	12.4 (6.3–NE)<br>17.9 (14.1–NE)	0.070
Bone metastasis	Yes (*n* = 3)<br>No (*n* = 45)	3.0 (1.4–NE)<br>4.9 (2.9–9.0)	0.424	11.7 (NE–NE)<br>17.9 (13.5–NE)	0.513
Asbestos exposure	Yes (*n* = 4)<br>No (*n* = 36)	7.8 (1.3–NE)<br>3.3 (2.4–8.8)	0.652	NE<br>14.1 (12.3–36.0)	0.322
Smoking history	Yes (*n* = 17)<br>No (*n* = 24)	3.3 (2.5–12.7)<br>4.9 (2.4–11.1)	0.893	17.9 (6.8–NE)<br>13.5 (11.7–NE)	0.971
Family history of cancer	Yes (*n* = 12)<br>No (*n* = 29)	4.8 (3.0–NE)<br>3.3 (2.4–9.0)	0.328	17.9 (13.5–NE)<br>13.5 (6.8–NE)	0.339
BAP1-associated malignancy history	Yes (*n* = 3)<br>No (*n* = 43)	2.0 (1.3–NE)<br>4.6 (2.9–7.0)	0.313	6.8 (4.3–NE)<br>14.6 (13.5–NE)	0.114
Prior anti-angiogenic therapy	Yes (*n* = 29)<br>No (*n* = 19)	3.3 (2.4–11.1)<br>6.3 (3.0–NE)	0.606	17.9 (12.4–NE)<br>14.6 (11.7–NE)	0.647
Prior bevacizumab exposure	Yes (*n* = 26)<br>No (*n* = 22)	3.3 (2.0–11.1)<br>6.5 (3.0–12.7)	0.323	17.9 (12.4–NE)<br>14.6 (11.7–NE)	0.663
Cross-line VEGFR-TKI exposure	Yes (*n* = 8)<br>No (*n* = 40)	3.5 (2.4–NE)<br>4.9 (3.0–9.0)	0.665	NE<br>14.6 (13.5–NE)	0.252
VEGFR-TKI agent	Apatinib (*n* = 12)<br>Anlotinib (*n* = 36)	7.9 (3.0–NE)<br>4.0 (2.0–7.0)	0.038	14.6 (6.3–NE)<br>17.9 (13.5–NE)	0.671

CI, confidence interval; OS, overall survival; PFS, progression-free survival; TKI, tyrosine kinase inhibitor; VEGFR, vascular endothelial growth factor receptor; NE, not estimable. Patients with missing data were excluded from the corresponding analysis.

Supplementary Table S3. Univariable Cox regression analyses for PFS and OS

Variable	Univariable Cox regression for PFS		Univariable Cox regression for OS	
	HR (95% CI)	*p* value	HR (95% CI)	*p* value
Age at VEGFR-TKI initiation (≥60 vs. <60 yr)	1.17 (0.62, 2.19)	0.633	1.46 (0.63, 3.40)	0.382
Male sex	1.20 (0.65, 2.21)	0.564	1.42 (0.62, 3.26)	0.413
Primary site (peritoneal vs. pleural)	0.54 (0.26, 1.15)	0.111	0.96 (0.39, 2.37)	0.936
Histologic subtype (non-epithelioid vs. epithelioid)	0.91 (0.37, 2.24)	0.845	0.57 (0.17, 1.89)	0.357
Regional lymph node metastasis	0.89 (0.48, 1.63)	0.701	0.74 (0.33, 1.67)	0.465
Distant organ metastasis	1.40 (0.75, 2.60)	0.292	1.44 (0.64, 3.25)	0.385
Lung metastasis	1.12 (0.51, 2.47)	0.779	0.66 (0.19, 2.26)	0.511
Liver metastasis	2.06 (0.62, 6.86)	0.238	3.02 (0.86, 10.63)	0.086
Bone metastasis	1.79 (0.41, 7.76)	0.438	1.96 (0.25, 15.36)	0.521
Asbestos exposure	0.79 (0.28, 2.25)	0.654	0.38 (0.05, 2.83)	0.344
Smoking history	1.05 (0.54, 2.01)	0.893	0.99 (0.43, 2.25)	0.972
Family history of cancer	0.69 (0.33, 1.45)	0.332	0.64 (0.25, 1.62)	0.346
BAP1-associated malignancy history	1.82 (0.55, 6.01)	0.325	2.58 (0.76, 8.79)	0.130
Prior anti-angiogenic therapy	1.18 (0.63, 2.18)	0.609	0.83 (0.37, 1.86)	0.649
Prior bevacizumab exposure	1.36 (0.74, 2.50)	0.328	0.84 (0.37, 1.88)	0.665
Cross-line VEGFR-TKI exposure	0.83 (0.37, 1.90)	0.667	0.50 (0.15, 1.69)	0.263
VEGFR-TKI agent (apatinib vs. anlotinib)	0.46 (0.21, 0.98)	0.043	1.21 (0.51, 2.87)	0.673

HR, hazard ratio; CI, confidence interval; OS, overall survival; PFS, progression-free survival; TKI, tyrosine kinase inhibitor; VEGFR, vascular endothelial growth factor receptor. PFS and OS were calculated from the initiation of oral VEGFR-TKI-containing therapy. Patients with missing data were excluded from the corresponding model.

Supplementary Table S4. Baseline and treatment characteristics according to VEGFR-TKI agent

**Variable**	**Category**	**Anlotinib (*n* = 36)**	**Apatinib (*n* = 12)**
Age at VEGFR-TKI initiation, years	Median (IQR)	58.6 (52.6–66.0)	61.0 (48.0–64.6)
Sex	Male	17 (47.2)	8 (66.7)
	Female	19 (52.8)	4 (33.3)
Primary site	Pleural	33 (91.7)	3 (25.0)
	Peritoneal	3 (8.3)	9 (75.0)
Histologic subtype	Epithelioid	17 (47.2)	5 (41.7)
	Biphasic	4 (11.1)	1 (8.3)
	Sarcomatoid	3 (8.3)	0 (0.0)
	Unknown	12 (33.3)	6 (50.0)
ECOG performance status	0	7 (19.4)	2 (16.7)
	1	19 (52.8)	5 (41.7)
	2	2 (5.6)	3 (25.0)
	Unknown	8 (22.2)	2 (16.7)
Prior systemic therapy lines	Median (range)	2 (1–5)	1 (1–2)
Prior anti-angiogenic therapy	Yes	26 (72.2)	3 (25.0)
	No	10 (27.8)	9 (75.0)
Prior bevacizumab exposure	Yes	25 (69.4)	1 (8.3)
	No	11 (30.6)	11 (91.7)
VEGFR-TKI treatment pattern	Monotherapy	15 (41.7)	3 (25.0)
	With ICB	5 (13.9)	1 (8.3)
	With chemotherapy	6 (16.7)	7 (58.3)
	With chemotherapy + ICB	10 (27.8)	1 (8.3)

Data are presented as *n* (%) unless otherwise indicated. Percentages are calculated within each VEGFR-TKI agent group. Age is reported as median (interquartile range), and prior systemic therapy lines as median (range). ECOG, Eastern Cooperative Oncology Group; ICB, immune checkpoint blockade; VEGFR-TKI, vascular endothelial growth factor receptor tyrosine kinase inhibitor. Data are presented descriptively; no formal between-group hypothesis testing was performed because of the small and uneven group sizes.
